# Pharmacognostic Evaluation and Antioxidant Profiling of Five Varieties of *Ribes nigrum* Grown in Romania

**DOI:** 10.3390/plants14111604

**Published:** 2025-05-24

**Authors:** Ruxandra Ștefănescu, Francisc Boda, Monica Sebestyen, Ioana Râșteiu, Eszter Laczkó-Zöld, Lénárd Farczádi

**Affiliations:** 1 Department of Pharmacognosy and Phytotherapy, Faculty of Pharmacy, George Emil Palade University of Medicine, Pharmacy, Science and Technology of Targu Mures, 540142 Targu Mures, Romania; ruxandra.stefanescu@umfst.ro (R.Ș.); eszter.laczko@umfst.ro (E.L.-Z.); 2 Department of General and Inorganic Chemistry, Faculty of Pharmacy, George Emil Palade University of Medicine, Pharmacy, Science and Technology of Targu Mures, 540142 Targu Mures, Romania; 3 Faculty of Pharmacy, George Emil Palade University of Medicine, Pharmacy, Science and Technology of Targu Mures, 540142 Targu Mures, Romania; monica.sebestyen@yahoo.ro (M.S.); ioana_ristei@yahoo.com (I.R.); 4 Chromatography and Mass Spectrometry Laboratory, Center for Advanced Medical and Pharmaceutical Research, George Emil Palade University of Medicine, Pharmacy, Science, and Technology of Targu Mures, 540142 Targu Mures, Romania; lenard.farczadi@umfst.ro

**Keywords:** *Ribes nigrum*, anthocyanin, pharmacognostical analysis

## Abstract

Blackcurrants (*Ribes nigrum* L.) are recognized for their rich anthocyanin content, contributing to their health-promoting properties. This study explored the phytochemical profiles and antioxidant activities of five blackcurrant varieties (Gofert, Tiben, Ceres, Ronix, and Ruben) cultivated in Mureș County, Romania. Using HPLC-DAD analysis, cyanidin-rutinoside was identified as the predominant anthocyanin in all varieties. Ruben stood out with the highest anthocyanin concentration and antioxidant activity, highlighting its potential for functional food and nutraceutical applications. The high anthocyanin content determined in these varieties suggests the influence of hybridization and unique regional growing conditions. Antioxidant activity, assessed through 2,2-Diphenyl-1-picrylhydrazyl (DPPH) and (2,2′-azino-bis(3-ethylbenzothiazoline-6-sulfonic acid)) (ABTS) assays, showed strong correlations with anthocyanin content, though polyphenolic levels did not consistently align with antioxidant efficacy. These findings emphasize the importance of tailoring blackcurrant cultivation to optimize bioactive compound production and support further exploration of their therapeutic potential.

## 1. Introduction

The blackcurrant (*Ribes nigrum* L.), is a shrub that belongs to the kingdom Plantae, class Magnoliopsida, order Saxifragaceae, and family Grossulariaceae. The genus *Ribes* contains between 140 and 150 species [1]. Blackcurrants are commercially significant fruits, with approximately 85% of global production concentrated in Europe, with Poland being the leading producer [2]. Various parts of the blackcurrant plant are utilized for their beneficial effects, including the fruits, flowers, leaves, and the oil extracted from its seeds [3].

Blackcurrants exhibit a distinctive bitter and astringent taste. Their pronounced acidity is mainly due to the high concentration of citric acid, while mandelic acid, present in lower quantities, also plays a contributory role in the fruit’s acidic profile [3]. The fruits contain an increased concentration of polyphenols, most of which are anthocyanins. The four main anthocyanins are delphinidin 3-*O*-rutinoside, cyanidin 3-*O*-rutinoside, delphinidin 3-*O*-glucoside, and cyanidin 3-*O*-glucoside [4].

Studies have shown that foods high in anthocyanins (e.g., currants, grapes) exhibit intense antioxidant effects. They are able to inactivate free radicals and stop the series of reactions that lead to oxidative damage of deoxyribonucleic acid. The first favorable effect of anthocyanins, demonstrated by in vitro and in vivo studies, was their impact on visual parameters [5,6,7]. Possible mechanisms include accelerating rhodopsin resynthesis, improving microcirculation, and modulating retinal enzymatic activity. According to epidemiological and experimental studies, foods containing anthocyanins are able to reduce inflammation and alleviate diseases associated with inflammation. The ability to reduce oxidative stress is thought to be closely linked to the beneficial effects of anthocyanins on cardiovascular disease. As additional mechanisms, anthocyanins have the ability to inhibit xanthine oxidase (XO), chelate Fe and Cu ions, inhibit prostaglandin E2 synthesis, and decrease the rate of endothelial nitric oxide (NO) synthesis [8]. All these effects are strictly correlated with the chemical composition. There are, however, important variations among different cultivars; therefore, standardization of the extracts is an important aspect, as well as identifying the best sources that have optimal composition.

The aim of this study was to comprehensively evaluate and compare the chemical composition and antioxidant capacity of five varieties of *Ribes nigrum* (Gofert, Tiben, Ceres, Ronix and Ruben) harvested from a crop in Mureș County, Romania, in order to evaluate how the phytochemical profiles differ among selected blackcurrant cultivars, and examine what implications these variations have for their antioxidant potential and further industrial applicability. Specifically, this study focuses on quantifying key phenolic compounds, including total polyphenols, anthocyanins, individual anthocyanins, flavonoids, and phenolic acids; it also assesses their contributions to antioxidant activity through DPPH and ABTS assays. In addition, this study includes the quantification of inorganic anions (chloride, phosphate, and sulfate) using ion chromatography, in order to provide a more comprehensive chemical characterization of the blackcurrant extracts. Multivariate analysis, including principal component analysis (PCA), was employed to explore the relationships between bioactive compounds and antioxidant potential. The characteristics of each variety are presented in Table 1.

## 2. Results

### 2.1. Total Polyphenol (TPC) and Total Anthocyanin (TA) Content 

**Figure 1:**
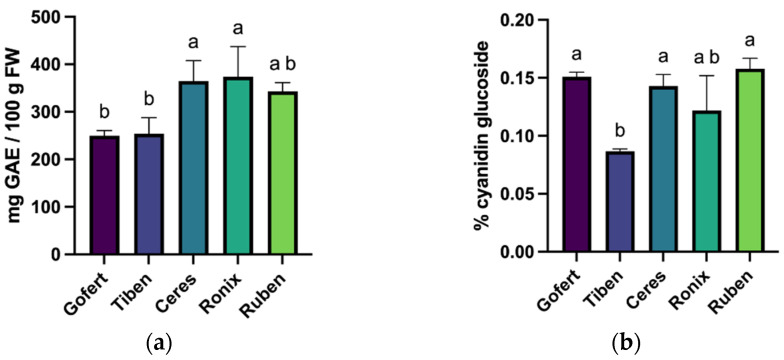
Total polyphenols (**a**), total anthocyanins (**b**); different letters in the columns represent statistically significant differences at *p* < 0.05.

The total polyphenol content varied significantly among the five blackcurrant varieties (F(4, 10) = 7.354, *p* = 0.005). Ceres and Ronix exhibited the highest polyphenol levels (364.7 ± 42.95 mg GAE/100 g FW and 373.7 ± 63.46 mg GAE/100 g FW, respectively), with no significant difference between them. In contrast, Gofert and Tiben presented significantly lower values (249.8 ± 10.97 mg GAE/100 g FW and 253.9 ± 33.95 mg GAE/100 g FW, while Ruben displayed intermediate content (Figure 1a).

Regarding total anthocyanin concentration (Figure 1b), Ruben and Gofert showed the highest levels, followed by Ceres and Ronix. The one-way ANOVA analysis indicated significant differences among varieties (F(4, 10) = 11.27, *p* = 0.001). Tiben had the lowest cyanidin-glucoside percentage, which was significantly different from all other varieties (*p* < 0.05). These results highlight notable differences in phenolic composition among the varieties, which may influence their respective antioxidant capacities.

### 2.2. HPLC-DAD Analysis of Anthocyanins

The identification and chromatographic separation of anthocyanins was performed, starting from the method described in the European Pharmacopoeia, 11th edition, in the monograph *Myrtilli fructus recentis extractum siccum raffinatum et normatum* [15]. The spectra with an absorption maximum (λmax) at 512 nm correspond to the cyanidin-type nucleus, and the compounds that have the absorption maximum at 521 nm correspond to the delphinidin-type nucleus. HPLC chromatograms of the analyzed extracts revealed the presence of three out of the five standard substances used. In the extracts of the Gofert, Tiben, Ceres, Ronix, and Ruben varieties, delphinidin-3-*O*-rutinoside, cyanidin-3-*O*-rutinoside, and cyanidin-3-*O*-glucoside could be identified.

**Figure 2:**
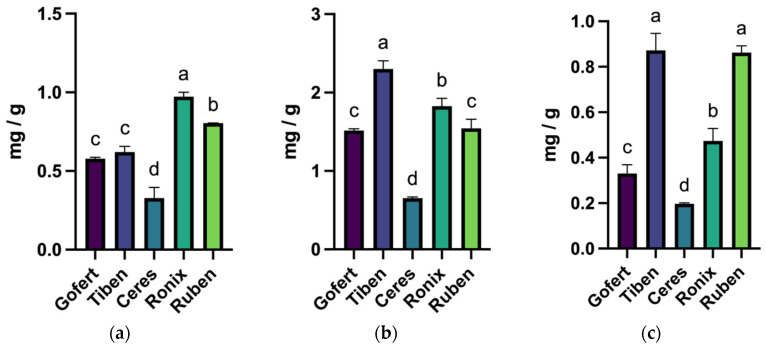
Concentration of delphinidin-3-*O*-rutinoside—DR (**a**), cyanidin-3-*O*-rutinoside—CR (**b**), and cyanidin-3-*O*-glucoside—CG (**c**) in the samples; different letters in the columns represent statistically significant differences among varieties.

As can be seen in Figure 2, the dominant anthocyanin is the cyanidin-3-*O*-rutinoside. The highest concentration of CR and CG was determined in the Tiben extracts, while the highest concentration of DR was determined in the Ronix extracts, with statistically significant differences compared to the other extracts (*p* < 0.05). The lowest content of individual anthocyanins was determined in the Ceres variety. For each quantified individual anthocyanin, the ANOVA analysis indicated significant differences among the following varieties: delphinidin rutinoside, F(4, 10) = 131.1, *p* < 0.0001; cyanidin-rutinoside, F(4, 10) = 154.6, *p* < 0.0001, and cyanidin-glucoside, F(4, 10) = 128.5, *p* < 0.0001.

### 2.3. UHPLC-PDA Analysis of Phenolic Compounds

The limit of quantification (LOQ) for each standard was established at 3.125 µg/mL, while the lower limit of quantification (LLOQ) was determined to be 1.5 µg/mL. The UHPLC-PDA analysis of *Ribes* extracts confirmed the presence of rutoside and caffeic acid in all analyzed extracts. However, catechin levels in the Ceres variety were below the LLOQ, whereas vanillic acid was under the LLOQ threshold in the Ronix variety. Furthermore, chlorogenic acid, gallic acid, and quercetin were all detected at concentrations below the LLOQ in every extract.

**Figure 3:**
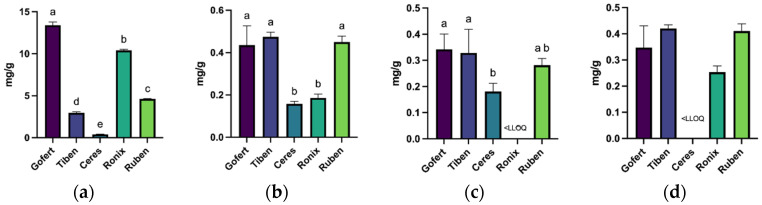
Concentration of individual phenolic compounds determined by UHPLC-PDA. (**a**) Rutin, (**b**) caffeic acid, (**c**) vanillic acid, (**d**) catechin. Different letters above columns indicate statistically significant differences at *p* < 0.05.

With respect to rutin content, the highest concentration was recorded in the Gofert variety (13.4 ± 0.39 µg/mL), while the lowest was observed in the Ceres variety (0.41 ± 0.003 µg/mL). Catechin was detected in all extracts except for Ceres, with no statistically significant differences among the remaining varieties. The concentration of caffeic acid was found to be comparable among the Gofert, Tiben, and Ruben varieties, yet it exhibited a statistically significant difference (*p* < 0.05) when compared to the Ceres and Ronix varieties (Figure 3). Regarding vanillic acid, the highest concentration was identified in the Gofert, Tiben, and Ruben varieties, while the lowest concentration was found in Ceres.

### 2.4. IC Analysis

**Figure 4:**
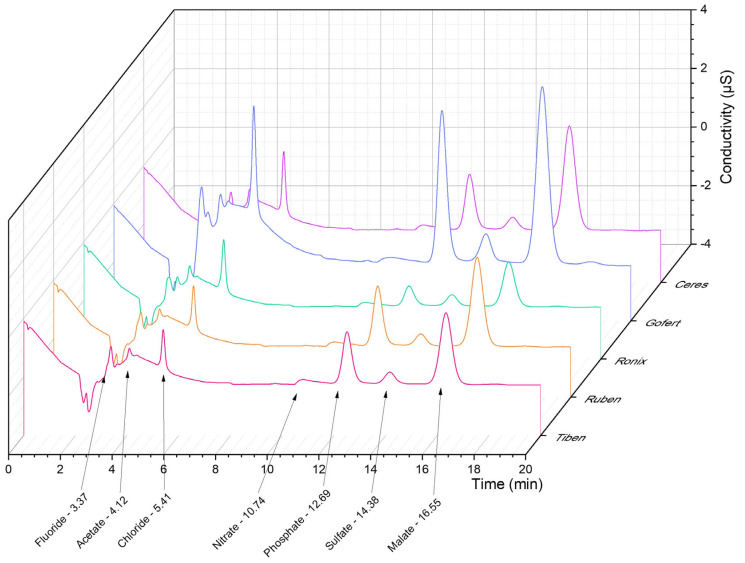
Composite chromatogram showing the anion content of the five different *Ribes* species, obtained via ion chromatography.

Ion chromatographic analysis allowed for the quantification of chloride (2.0–3.5 ppm), phosphate (6.0–29.5 ppm), and sulfate (1.8–3.3 ppm) ions (Figure 4). Among the tested samples, Gofert extract presented a significantly higher phosphate content (29.5 ppm), while the smallest amount of phosphate could be found in the Ronix extract (6.0 ppm). The presence of fluoride and nitrate ions was also observed in all samples, but was under the limit of quantification. Using the spike method, we identified the retention times (Rt) for the peaks corresponding to acetate (Rt = 4.12 min) and malate (Rt = 16.55 min) ions. Ascorbate and citrate ions did not provide a measurable signal under the conditions used for the analysis, possibly due to their mass-to-charge ratio, inadequate for conductometric determination.

### 2.5. Determination of Antioxidant Activity Through DPPH and ABTS Free Radical Scavenging Assay

The antioxidant evaluation of the five extracts using DPPH revealed that Tiben has the highest IC_50_, which is statistically significantly different from the other extracts, meaning that it has the lowest antioxidant activity (Table 2). The ABTS analysis revealed that the highest IC_50_ was obtained with Gofert and Ronix extracts.

### 2.6. Pearson’s Correlation and Principal Component Analysis (PCA)

**Figure 5:**
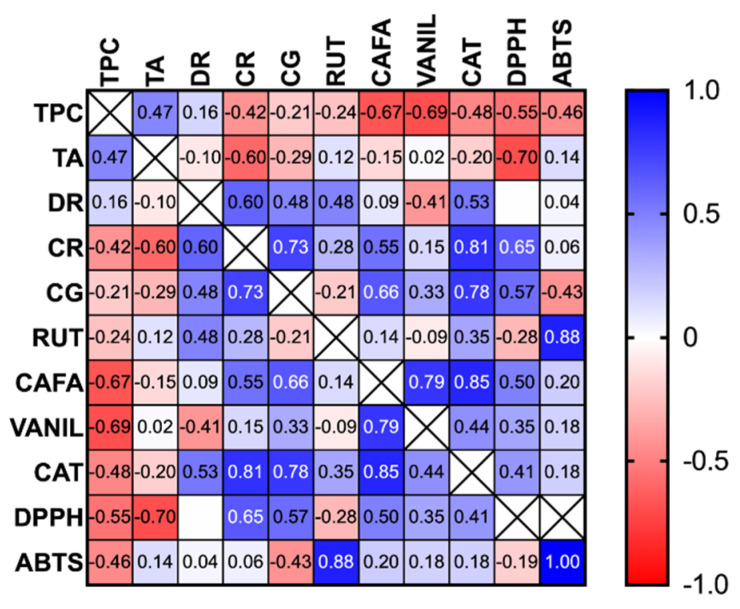
Heatmap of Pearson’s correlations. TPC—total polyphenolic content, TA—total anthocyanins, DR—delphinidin rutinoside, CR—cyanidin-rutinoside, CG—cyanidin-glucoside, RUT—rutin, CAFA—caffeic acid, VANIL—vanillic acid, CAT—catechin.

There is a strong negative correlation between the total anthocyanin content and antioxidant activity determined with DPPH (r = −0.7, *p* < 0.005). Also, negative correlations were observed between TPC and antioxidant activity determined with both DPPH and ABTS. A positive correlation between individual anthocyanins (delphinidin rutinoside, cyanidin-rutinoside, and cyanidin-glucoside) and catechin was observed (Figure 5). The correlation was stronger between catechin and cyanidin glycosides compared to delphinidin, probably due to the biosynthetic pathway.

**Figure 6:**
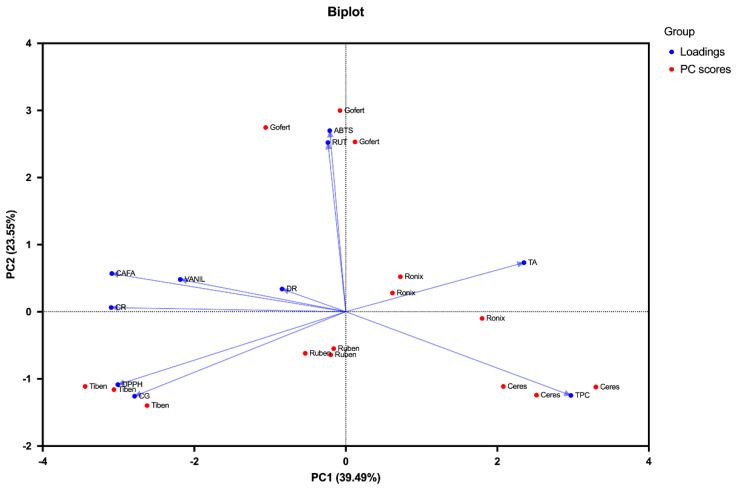
Bi-plot analysis of phytochemical and antioxidant characteristics.

Principal component analysis (PCA) was conducted to explore the relationships among the chemical composition and antioxidant activities of the five extracts. The first three principal components (PCs) accounted for 83.53% of the total variance, with PC1 explaining 39.49%, PC2 accounting for 23.55%, and PC3 contributing 20.49%. The PCA biplot (PC1 vs. PC2) revealed clear differentiation among the varieties (Figure 6). Ceres was distinctly associated with high TPC and TA, aligning strongly with the positive axis of PC1. This suggests that Ceres may offer superior antioxidant potential, attributed to its elevated polyphenol and anthocyanin content. Tiben was located on the negative axis of both PC1 and PC2, associated with cyanidin-3-*O*-glucoside (CG), cyanidin-3-*O*-rutinoside (CR), and higher DPPH IC_50_ values, suggesting a lower overall antioxidant efficiency compared to the other varieties. Ronix and Ruben occupied intermediate positions in the biplot. Ronix was closely associated with TA, while Ruben was centrally positioned, suggesting a balanced phenolic composition and antioxidant profile without dominance of specific compounds.

## 3. Discussion

This study aimed to evaluate and compare the phytochemical profile and antioxidant activity of five *Ribes nigrum* (blackcurrant) varieties cultivated in Mureș County, Romania. The research topic was chosen based on the global interest in so-called superfoods, as well as the relatively limited popularity and utilization of blackcurrant fruits compared to other commonly consumed berries such as blueberries and strawberries [16,17].

The results obtained in this study demonstrate significant variation in both total polyphenol content and total anthocyanin content among the five analyzed varieties. Such variability is consistent with previous research, highlighting the influence of genetic factors on the phytochemical composition of blackcurrant fruits. The polyphenol content in the fruits was significantly higher compared to the concentration obtained by Laczkó-Zöld et al. and Karaklajic-Stajic [18,19]. Following the determination of total anthocyanins, expressed as cyanidin-3-*O*-glucoside, it can be observed that the Gofert, Ceres, and Ruben varieties present the highest levels, with no statistically significant differences among them. Anthocyanins, particularly cyanidin-3-*O*-glucoside, are among the most potent antioxidant compounds found in blackcurrants, contributing significantly to their nutritional and functional properties [19,20]. Our study demonstrated that there is a strong correlation between TA and the antioxidant activity determined with the DPPH method. No correlations were noticed between the DPPH and ABTS results, but this trend is not uncommon since previous studies have demonstrated notable differences between the two methods. These differences can be the result of several factors, such as chemical structure, the number of hydroxyl and methoxy groups, pH, solvent, the speed of reaction, etc. [21,22]. Sample preservation plays a critical role in ensuring the accuracy and reproducibility of polyphenol quantification. In this study, plant materials were stored at −20 °C immediately after harvesting, a commonly used method in phytochemical research. However, we recognize that immediate freezing in liquid nitrogen followed by lyophilization represents a superior approach for preserving thermolabile and oxidation-prone compounds, such as polyphenols. At the time of the study, however, no such treatment was possible, which we acknowledge as a limitation of our protocol.

As can be seen in Figure 2, the main determined anthocyanins in some varieties are delphinidin-3-*O*-rutinoside and cyanidin-3-*O*-rutinoside; the results are consistent with the results obtained by Slimestad and Solheim [23], as well as with the results obtained by Paunovic et al. regarding the quantitatively dominant compounds [24]. In contrast to the results obtained by Paunovic et al., the concentrations of anthocyanins determined in the present study are almost double [24]. Also, comparing the concentration of individual CG in all the varieties with the results obtained by Laczkó-Zöld et al., we can conclude that the varieties analyzed in this study contained much higher concentrations [18]. However, Maatta et al. obtained similar results to the present study [25]. Another study carried out on the stability and composition of anthocyanins in *R. nigrum* fruits concluded that the major anthocyanin is represented by cyanidin-rutinoside, with the mention that the concentration data may change depending on the year of cultivation [26].

**Figure 7:**
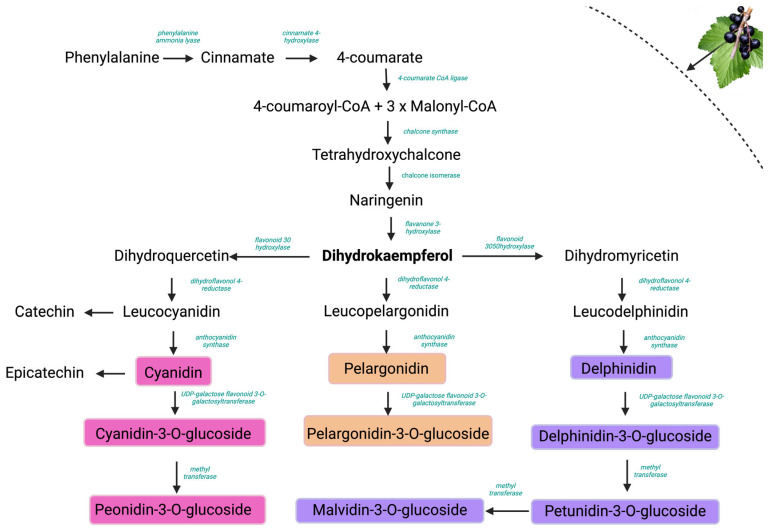
Biosynthetic pathway of anthocyanin formation.

Although different reports suggest that there is a positive correlation between the occurrence of quercetin glycosides and cyanidin glycosides, and a negative correlation between quercetin glycosides and delphinidin glycosides, our results indicate the opposite. This result may reflect differences in the specific biosynthetic pathways of the analyzed varieties or cultivar-specific metabolic regulations [27]. Delphinidin and cyanidin, along with their glycosides, are formed through different biosynthetic pathways, starting from dihydrokaempferol, as seen in Figure 7. F3′H (flavonoid 3′-hydroxylase) favors cyanidin biosynthesis, whereas F3′5′H (flavonoid 3′,5′-hydroxylase) leads to delphinidin derivatives. The competition between these enzymes for shared intermediates can shift metabolic flux toward either the cyanidin or delphinidin branch. In parallel, quercetin glycosides are formed via the flavonol synthase (FLS)-mediated pathway from dihydroquercetin. A shift in gene expression favoring F3′5′H and FLS over F3′H could be an explanation for the observed association between delphinidin and quercetin glycosides in our varieties [28,29].

Additionally, anthocyanin accumulation may be affected by environmental conditions. The environmental factors that increase anthocyanin content in fruits are light exposure and a combination of light and low temperatures [19]. The synthesis of anthocyanins is decreased by high temperatures (30–35 °C), excess nitrogen fertilizer, and shade [30,31]. The varieties in this study were cultivated under uniform agronomic conditions, effectively minimizing environmental variability as a factor. Our results denote the same conclusion reached by Parkar et al. [32].

The determination of total anthocyanins, expressed as cyanidin-3-*O*-glucoside, further reinforces the prominence of the Gofert, Ceres, and Ruben varieties in terms of anthocyanin accumulation. The absence of statistically significant differences among these varieties suggests a potential threshold beyond which anthocyanin accumulation stabilizes, possibly due to saturation effects or feedback inhibition within the biosynthetic pathway. These findings could be relevant for breeding programs aiming to enhance anthocyanin content for nutraceutical or industrial applications.

As can be seen in Table 3, the concentrations of total polyphenols and total anthocyanins exhibit considerable variability, which can be attributed to differences in geographical origin and potentially to cultivation conditions. Factors such as soil composition, climate, altitude, and agricultural practices—including irrigation, fertilization, and harvesting methods—can significantly influence the biosynthesis and accumulation of these compounds in plants.

The concentration of phenolic compounds observed in this study aligns with previously published findings. However, the levels of caffeic acid detected were notably higher than those reported by Maatta et al. [25]. These discrepancies can be attributed to the influence of geographical and environmental factors on the metabolic pathways involved in phenolic compound biosynthesis. Given the well-documented variability induced by such external conditions, these differences are both expected and justified.

The positive correlation observed between individual anthocyanins (delphinidin rutinoside, cyanidin-rutinoside, and cyanidin-glucoside) and catechin suggests a synergistic contribution of these compounds to the overall antioxidant activity of the blackcurrant extracts. Both anthocyanins and catechins have been well-documented for their ability to neutralize free radicals, primarily due to their hydroxyl-rich structures that facilitate electron donation [5,36].

The results obtained for the antioxidant activity of the five *Ribes* extracts using the DPPH and ABTS methods highlight significant differences between the varieties. In the DPPH assay, the extract from the Tiben variety showed the lowest antioxidant activity, which was significantly different compared to the other extracts (*p* < 0.05). In contrast, the strongest antioxidant activity (i.e., the lowest IC_50_ values) was observed in the extracts from the Ceres, Ronix, and Gofert varieties, with no statistically significant differences among them, suggesting a comparable efficiency in inhibiting DPPH radicals. The ABTS assay provided complementary results, but with a better differentiation among the tested varieties. The lowest IC_50_ values, corresponding to the highest antioxidant activity, were recorded for the Ruben and Ceres extracts. Conversely, Gofert and Ronix exhibited the highest IC_50_ values, indicating weaker antioxidant activity in this antioxidant test. These variations in the antioxidant activity suggest that the phytochemical profile of each extract contributes differently to this activity, and that cultivar selection is a key factor in obtaining extracts with high therapeutic potential.

Ion chromatographic profiling revealed significant variability in the anion composition among the five *Ribes* extracts, despite identical soil conditions for all samples. Gofert extract stood out with the highest concentrations of chloride, phosphate, and sulfate, suggesting a distinct mineral uptake pattern or enhanced accumulation ability compared to other varieties. As Gofert had one of the highest total anthocyanin concentrations as well, this observation may suggest a potential link between elevated inorganic ion levels and enhanced anthocyanin biosynthesis. In contrast, Ronix extracts showed the lowest values for several ions, including phosphate, chloride, and sulfate, while expressing a high total polyphenol level. Tiben extracts, with lower anthocyanin and polyphenol contents, also exhibited the lowest chloride and sulfate levels, further reinforcing the observation that mineral nutrient availability may contribute to the secondary metabolite biosynthesis.

Fluoride and nitrate were detected in all samples but remained within a narrow range, with fluoride concentrations between 0.55 and 0.59 ppm and nitrate between 1.29 and 1.33 ppm, indicating a relatively uniform environmental exposure or regulatory uptake across varieties. Nitrite and bromide remained below the limit of detection (LOD) in all samples. Organic acid profiling confirmed the presence of acetate and malate, while ascorbate and citrate were undetectable—likely due to limitations of the conductometric detection method. These results emphasize that the ionic composition of *Ribes* extracts—particularly phosphate and sulfate levels—may play a relevant role in their phytochemical and functional properties, highlighting the importance of including inorganic ion analysis as a chemotaxonomic tool or a quality control marker for extract standardization.

The differentiation among varieties revealed by PCA provides valuable insights for breeding programs aimed at enhancing the health-promoting properties of *Ribes nigrum*.

While the current study provides important insights into the phytochemical diversity and antioxidant potential of blackcurrant varieties, it is limited to in vitro assays and chemical composition. Future research should explore the bioavailability and in vivo effects of these compounds, as well as the impact of agronomic practices, post-harvest processing, and storage conditions on their stability and efficacy.

## 4. Materials and Methods

### 4.1. Plant Collection

The harvesting of the 5 species of blackcurrant variants was carried out from a culture in Mureș County. The culture is located on the right bank of the Târnava Mică River, near DN13A.

The blackcurrants were harvested according to the period in which the fruits reached maturity, as follows: the fruits from the Gofert variety were harvested at the end of June; the fruits of the Ronix variety were harvested at the beginning of July; and the fruits from the Ruben, Tiben, and Ceres varieties were harvested in the middle of July. About 0.5 kg of fruits of each variety were harvested, which were subsequently frozen at −20 °C and stored in the freezer until the extraction process. Botanical identification was performed and confirmed by the academic staff from the Discipline of Pharmacognosy and Phytotherapy, based on morphological characteristics and cultivar information provided by the grower. From each variety, a voucher specimen was deposited at the Department of Pharmacognosy and Phytotherapy, from the Faculty of Pharmacy of the George Emil Palade University of Medicine, Pharmacy, Science and Technology of Târgu Mureș, Romania (accession numbers: CT-RN-Go20, CT-RN-Ro20, CT-RN-Ru20, CT-RN-Tb20, CT-RN-Cs20).

### 4.2. Chemicals

All the solvents used in this experiment were of HPLC grade with a purity > 99.9%. The following standards were used for the identification and quantification of anthocyanins: cyanin chloride, cyanidin 3-*O*-rutinoside chloride, and delphindin-3-*O*-rutinoside chloride were purchased from Roth (Carl Roth GmbH, Karlsruhe, Germany); cyanidin 3-*O*-glucoside was purchased from Applichem Panreac (Darmstadt, Germany) and delphinidin chloride, rutin, caffeic acid, chlorogenic acid, quercetin, gallic acid, and vanillic acid were purchased from Sigma-Aldrich (Darmstadt, Germany). The ultrapure water was obtained using a Direct-Q system (Millipore, Bedford, MA, USA).

### 4.3. Extracts Preparation

About 40 g of frozen fruit from each variety was crushed in a mortar with the help of a pestle; this operation was repeated for each variety. Moreover, 10 g of crushed fruit from each variety was weighed on an analytical balance (Kern ABJ 220-4M, Kern & Sohn GmbH, Balingen, Germany) and extracted with 100 mL of 0.1% HCl in methanol for 30 min in a Nahita 626 ultrasonic water bath (Auxilab, Beriáin, Spain). The extracts were filtered through a Whatman No. 2 filter paper and were brought to scale in 100 mL volumetric flasks. Post-preparation, the extracts were stored in the fridge at 4 °C, in tinted glass bottles. Prior to the experiments, the extracts were allowed to reach room temperature and were shaken thoroughly.

### 4.4. Total Polyphenol Content (TPC) Determination

For the TPC determination, a micro-method was used [37,38,39]. Briefly, 40 μL of the sample was mixed with 3160 μL of distilled water; then, 200 μL of Folin–Ciocalteu reagent (Sigma-Aldrich, Darmstadt, Germany) was added, and the samples were vortexed thoroughly (1 min). After mixing the samples with the FC reagent, 600 μL of sodium carbonate solution (200 g/L) was added; the samples were shaken and left to stand for 30 min at room temperature. The absorbance was measured at 765 nm using a UV-Vis SPECORD spectrophotometer (Analytic Jena, Jena, Germany). The calibration curve of gallic acid was performed using the same conditions. Total polyphenol content was expressed as mg gallic acid equivalents (GAEs)/100 g of fresh weight (FW) using the following equation based on the calibration curve: y = 26.394x + 0.0516 (R^2^ = 0.967).

### 4.5. Total Anthocyanin Content (TAC)

For TAC content, the method from the European Pharmacopoeia 8th Edition was used. The extracts were diluted 50× with a solution of 0.1% HCl in methanol, and the absorbance was read at 528 nm. The results were expressed as % cyanidin-3-*O*-glucoside and were calculated with the following formula: C = (A × 5000)/(718 × m), where A is the absorbance of the test solution, 718 is the specific absorbance of cyanidin 3-*O*-glucoside, and m is the g of herbal product used for the extract.

### 4.6. HPLC-DAD Analysis of Anthocyanins

Anthocyanin quantification was performed according to the method described in the European Pharmacopoeia 7th edition. According to the Eur. Ph. for HPLC analysis, a column with the following dimensions was used: l = 0.250 m, Ø = 4.6 mm, and the recommended mobile phase was A: anhydrous formic acid: water (8.5:91.5, *V*/*V*) and B: formic acid anhydrous: acetonitrile: methanol: water (8.5:22.5:22.5:41.5, *V*/*V*/*V*/*V*), with the following gradient: 0–35 min—A: 93–75%; 35–45 min—A: 75–35%, 45–46 min—A: 35–0%, 46–50 min—A: 0%. Since the column available for this determination was shorter than that recommended by the Ph. Eur., the gradient was slightly modified. The identification of the compounds in the extracts was conducted based on the spectra and retention times. The following compounds were used for the standard mixture: cyanidin (Rt = 3.2 min), delphinidin-3-*O*-rutinoside (Rt = 3.94 min), cyanidin-3-*O*-rutinoside (Rt = 5.7 min), cyanidin-3-*O*-glucoside (Rt = 5.23 min), and delphinidin (Rt = 6.78 min). Analytical experiments were performed on a Merck HPLC system equipped with a photodiode array detector. A Luna^®^ C18 column (100 × 4.6 mm, 3 µm, Phenomenex) was used. The mobile phase consisted of anhydrous formic acid–water (8.5:91.5 *V*/*V*) as solvent A and anhydrous formic acid–acetonitrile–methanol–water (8.5:22.5:22.5:41.5 *V*/*V*/*V*/*V*) as solvent B. The following gradient elution was used: 0–35 min 5–25% B, 35–45 min 25–65% B, 45–46 min 65–100% B, and 46–50 min 100% B, at a low rate of 1 mL/min. The injection volume was 10 µL, and detection was at 535 nm. The peaks were recorded at 270, 280, and 370 nm. 

### 4.7. UHPLC-PDA Analysis of Phenolic Compounds

The identification of phenolic compounds was performed using a UPLC Flexar FX-10 Perkin Elmer system equipped with a binary pump, in-line degasser, column thermostat, autosampler, and a Flexar FX-PDA UHPLC detector and a Luna^®^ C18 column (100 × 4.6 mm, 3 µm, Phenomenex, Torrance, CA, USA). A gradient elution with 0.1% formic acid as phase A and acetonitrile as phase B was used according to a previously published method by Tanase et al. [40]. Gallic acid, vanillic acid, quercetin, catechin, chlorogenic acid, caffeic acid, and rutin were used as standards. The standard solutions were prepared by dissolving the standards in methanol. A calibration curve for each standard was constructed in the concentration range of 1.5–200 µg/mL, using 7 different concentrations.

### 4.8. Ion Chromatography Analysis

The measurements were conducted using a Dionex ICS-3000 ion chromatography system (Thermo Fisher Scientific, Waltham, MA, USA) with suppressed conductivity detection. Anions were separated on a Dionex IonPac AS23 (250 × 4 mm) analytical column. The experiment was conducted following the manufacturer’s recommended method, with interlaboratory validation confirming its applicability for our samples. The applied analytical conditions were as follows: analysis time of 20 min; isocratic elution with an eluent composed of 0.45 mM of sodium carbonate and 0.08 mM of sodium bicarbonate; an eluent flow rate of 1.2 mL/min; the column type was Dionex IonPac AS23 (250 × 4 mm); the column temperature was 25 °C; and the injected sample volume was 50 µL. The Dionex Combined Seven Anion Standard II solution (Thermo Fisher Scientific, Waltham, MA, USA) was used as a reference for the determination of bromide, chloride, fluoride, nitrite, nitrate, phosphate, and sulfate. Analytical grade disodium malate, sodium acetate, sodium ascorbate, and potassium citrate (Merck, KGaA, Darmstadt, Germany) were used to identify corresponding peaks. All measurements were performed in triplicate. Data acquisition and processing were performed using the Chromeleon 6.80 Chromatography Management System (Thermo Fischer Scientific, Waltham, MA, USA).

### 4.9. Antioxidant Activity Through the DPPH Free Radical Scavenging Assay

The antioxidant activity of the extracts was determined using a stock solution of 1 mM 2,2-Diphenyl-1-picrylhydrazyl (DPPH) (Sigma-Aldrich, Darmstadt, Germany). A 150 µL sample (containing different concentrations of extract) was mixed with 2600 µL of DPPH solution. The mixture was well shaken, and after 30 min, the absorbance was read at 517 nm. The DPPH radical scavenging activity was determined by using the following formula: IC% = [(A_0_ − A_1_)/A_0_] × 100, where IC% is the percent of inhibition, A_0_ is the absorbance of the DPPH solution, and A_1_ is the absorbance of the sample. Ascorbic acid was used as the positive control.

### 4.10. Antioxidant Activity Through the ABTS Free Radical Scavenging Assay

The antioxidant activity of the Ribes extracts was analyzed with a stock solution of 2,2′-azino-bis(3-ethylbenzothiazoline-6-sulfonic acid (ABTS) (Roche Diagnostics GmbH, Mannheim, Germany), which was prepared 12 h before the determination. Moreover, 50 μL of the sample was mixed with 50 μL of distilled water using a 96-well plate; for each sample, 3 wells were used. The mixtures were diluted using the method of successive dilutions. After 5 min, the samples were analyzed using the Epoch microplate UV-VIS spectrophotometer (BioTek Instruments Inc., Winooski, VT, USA). The free radical scavenging activity was determined by calculating IC%, but in this case, A0 was the absorbance of the ABTS solution. Trolox was used as the positive control.

### 4.11. Statistical Analysis

The statistical analysis was performed using GraphPad Prism 9.0 statistical package software. The normality of distribution was assessed using the Kolmogorov–Smirnov test. All parameters were distributed normally. The results were expressed as the mean ± standard deviation of three replicates. To compare the datasets, ANOVA one-way analysis of variance was performed, and the Tukey multiple comparison test was used as a post hoc test. To analyze the association between the chemical composition and the free radical scavenging activity of the samples (DPPH and ABTS assays), Pearson’s correlation test was used. Principal component analysis (PCA) was performed to explore patterns and correlations among the chemical composition and antioxidant activities of the extracts. Values of *p* < 0.05 were considered statistically significant.

## 5. Conclusions

This study emphasizes the importance of region-specific research in understanding the bioactive compound variability in blackcurrant varieties. Significant variability was observed among the varieties in terms of total polyphenol content, total anthocyanin content, and individual phenolic compound concentrations. Among them, the Ceres variety exhibited high levels of both TPC and TA; however, its concentration of individual anthocyanins was comparatively lower than that of other varieties. In contrast, Gofert extracts were characterized by elevated levels of TA and individual phenolics. Tiben had the highest concentration of cyanidin glycosides, while in Ronix, a predominance of anthocyanin glycosides, particularly those conjugated with rutinoside, was observed.

These findings highlight the unique metabolic profiles inherent to each variety and emphasize the complexity of secondary metabolite biosynthesis in *Ribes nigrum*. Given these distinct phytochemical characteristics, it is challenging to identify a single variety as superior in terms of overall bioactive compound composition. Instead, each cultivar offers specific phytochemical advantages, underscoring the need for targeted selection based on the desired functional or nutritional application. This study contributes to the ethnobotanical knowledge of blackcurrants by supporting their traditional uses through the complex pharmacognostical analysis. Future studies should include multi-year harvests and multiple geographical sites in order to evaluate the stability of the polyphenolic compounds over time and in different environmental conditions.

## Figures and Tables

**Table 1:** Characteristics of the varieties of Ribes nigrum included in the present study.

Variety	Parents and Origin	Characteristics	References
Gofert	Golubka × Fertodi-*1* Origin: Poland	It is an early-maturing and high-yielding variety, exhibiting enhanced resistance to pests. The fruits are of medium size and are characterized by low acidity, while both anthocyanin and vitamin C levels are high.	[9]
Tiben	Titania × Ben Nevis Origin: Poland	The shrubs reach medium size, as do the fruits, which are also of moderate dimensions. This variety demonstrates strong resistance to diseases. The vitamin C content is modest.	[10,11]
Ceres	Pavlinka × Pilot Origin: Poland	This variety is characterized by medium-sized shrubs and large fruits, with enhanced resistance to frost. The fruit harvesting period is late, typically occurring in mid-July. The ‘Ceres’ variety is distinguished by its low acidity.	[12]
Ronix	Tsema × Kantata Origin: Romania	The shrub exhibits vigorous growth and large dimensions, while the berries are resistant to cracking. This semi-early variety shows good disease resistance and is notable for its high vitamin C content as well as high acidity.	[13]
Ruben	Bieloruskoja Slodkaja *×* Ben Lomond Origin: Poland	It is a high-yielding variety, characterized by medium to large fruits, with notable resistance to various diseases and tolerance to harsh winter conditions.	[11,14]

**Table 2:** The values of IC_50_ for the antioxidant activity of the *Ribes* extracts.

Variety	IC_50_ DPPH (µg/mL)	IC_50_ ABTS (µg/mL)
Gofert	1.95 ± 0.26 ^b^	3.06 ± 0.14 ^a^
Tiben	2.78 ± 0.07 ^a^	0.71 ± 0.10 ^c^
Ceres	1.89 ± 0.21 ^b^	0.58 ± 0.10 ^c^
Ronix	1.96 ± 0.25 ^b^	1.39 ± 0.37 ^b^
Ruben	1.98 ± 0.12 ^b^	0.56 ± 0.07 ^c^

The results are expressed as mean ± standard deviation; IC_50_—half maximal inhibitory concentration, ABTS—2,2′-azino-bis(3-ethylbenzothiazoline-6-sulfonic acid; DPPH—2,2-diphenyl-1-picrylhydrazyl; different letters in the same column indicate statistically significant differences at p < 0.05.

**Table 3:** TPC and TAC determined in previous studies in *Ribes nigrum* fruits collected from different geographical origins.

Origin of the Analyzed Samples	TPC (mg GAE/100 g FW)	TAC (%)	References
Finland	-	0.27–0.67	[27]
Romania	110–195	0.18–0.32	[18]
Serbia	-	0.20–0.31	[24]
Serbia	164	0.13	[19]
Belarus	354–408	0.23–0.27	[28]
Estonia	324–506	0.19–0.35	[28]
Finland	428–534	0.27–0.36	[28]
Latvia	358	0.24	[28]
Lithuania	328–470	0.20–0.32	[28]
Norway	290–634	0.18–0.47	[28]
Poland	362–515	0.24–0.35	[28]
Russia	460	0.31	[28]
Scotland	469–613	0.30–0.44	[28]
Sweden	343–529	0.22–0.37	[28]
Ukraine	424	0.29	[28]
Italy	530–888	0.15–0.28	[33]
Poland	163–195 *	0.14–0.17 *	[34]
Slovenia	452–662	-	[35]

Only studies where the results were reported based on fresh weight are included in the present table. * The results are expressed as the sum of individual compounds.

## Data Availability

The original contributions presented in the study are included in the article, further inquiries can be directed to the corresponding author.

## Abbreviations

The following abbreviations are used in this manuscript:

ABTS	2,2′-azino-bis(3-ethylbenzothiazoline-6-sulfonic acid)
CAFA	caffeic acid
CAT	catechin
CG	cyanidin-3-*O*-glucoside
CR	cyanidin-3-*O*-rutinoside
DPPH	2,2-Diphenyl-1-picrylhydrazyl
DR	delphinidin-3-*O*-rutinoside
FW	fresh weight
GAE	gallic acid equivalent
IC_50_	concentration of inhibitor resulting in 50% inhibition
LLOQ	lower limit of quantification
LOQ	limit of quantification
NO	nitric oxide
PCA	principal component analysis
RUT	rutin
TPC	total polyphenolic content
VANIL	vanillic acid
XO	xanthine oxidase

## References

[1] Assessment Report on *Ribes nigrum* L., Folium. 29. https://www.ema.europa.eu/en/documents/herbal-report/draft-assessment-report-ribes-nigrum-l-folium_en.pdf.

[2] Global-BC-Production-2022-2024.Pdf. https://www.blackcurrant-iba.com/wp-content/uploads/2024/07/Global-BC-Production-2022-2024.pdf.

[3] Nayik G.A., Gull A. (2020). Antioxidants in Fruits: Properties and Health Benefits.

[4] Lee Y., Lee J.-Y. (2019). Blackcurrant *(Ribes nigrum*) Extract Exerts an Anti-Inflammatory Action by Modulating Macrophage Phenotypes. Nutrients.

[5] Khoo H.E., Azlan A., Tang S.T., Lim S.M. (2017). Anthocyanidins and Anthocyanins: Colored Pigments as Food, Pharmaceutical Ingredients, and the Potential Health Benefits. Food Nutr. Res..

[6] Ştefănescu R., Vari C., Imre S., Huţanu A., Fogarasi E., Todea T., Groşan A., Eşianu S., Laczkó-Zöld E., Dogaru M. (2018). *Vaccinium* Extracts as Modulators in Experimental Type 1 Diabetes. J. Med. Food.

[7] Speciale A., Molonia M.S., Muscarà C., Cristani M., Salamone F.L., Saija A., Cimino F. (2024). An Overview on the Cellular Mechanisms of Anthocyanins in Maintaining Intestinal Integrity and Function. Fitoterapia.

[8] Reis J.F., Monteiro V.V.S., de Souza Gomes R., do Carmo M.M., da Costa G.V., Ribera P.C., Monteiro M.C. (2016). Action Mechanism and Cardiovascular Effect of Anthocyanins: A Systematic Review of Animal and Human Studies. J. Transl. Med..

[9] Pluta S., Żurawicz E. (2009). The Last Twenty Years Of Blackcurrant (*Ribes nigrum* L.) Breeding Work In Poland. Acta Hortic..

[10] Pluta S., Zurawicz E. (2002). “Tiben” And “Tisel”—New Blackcurrant Cultivars Released In Poland. Acta Hortic..

[11] Pluta S., Żurawicz E. (2008). Production Value And Suitability Of New Polish Blackcurrant Cultivars For Mechanical Fruit Harvesting. Acta Hortic..

[12] Lanham P.G., Brennan R.M., Hackett C., McNicol R.J. (1995). RAPD Fingerprinting of Blackcurrant (*Ribes nigrum* L.) Cultivars. Theoret. Appl. Genet..

[13] Braniște N. (2007). Soiuri de pomi, arbuști fructiferi și căpșuni create în România. Paralela.

[14] Pluta S., Żurawicz E. (2004). “Ores” And “Ruben”—New Blackcurrant Cultivars Bred In Poland. Acta Hortic..

[15] Council of Europe (2019). European Pharmacopoeia.

[16] Mattioli R., Francioso A., Mosca L., Silva P. (2020). Anthocyanins: A Comprehensive Review of Their Chemical Properties and Health Effects on Cardiovascular and Neurodegenerative Diseases. Molecules.

[17] Untea A.E., Oancea A.-G., Vlaicu P.A., Varzaru I., Saracila M. (2024). Blackcurrant (Fruits, Pomace, and Leaves) Phenolic Characterization before and after In Vitro Digestion, Free Radical Scavenger Capacity, and Antioxidant Effects on Iron-Mediated Lipid Peroxidation. Foods.

[18] Laczkó-Zöld E., Komlósi A., Ülkei T., Fogarasi E., Croitoru M., Fülöp I., Domokos E., Ştefănescu R., Varga E. (2018). Extractability of Polyphenols from Black Currant, Red Currant and Gooseberry and Their Antioxidant Activity. Acta Biol. Hung..

[19] Karaklajic-Stajic Z., Tomic J., Pesakovic M., Paunovic S.M., Stampar F., Mikulic-Petkovsek M., Grohar M.C., Hudina M., Jakopic J. (2023). Black Queens of Fruits: Chemical Composition of Blackberry (*Rubus* Subg. *Rubus* Watson) and Black Currant (*Ribes nigrum* L.) Cultivars Selected in Serbia. Foods.

[20] Kierońska E., Skoczylas J., Dziadek K., Pomietło U., Piątkowska E., Kopeć A. (2024). Basic Chemical Composition, Selected Polyphenolic Profile and Antioxidant Activity in Various Types of Currant (*Ribes* spp.) Fruits. Appl. Sci..

[21] Wołosiak R., Drużyńska B., Derewiaka D., Piecyk M., Majewska E., Ciecierska M., Worobiej E., Pakosz P. (2022). Verification of the Conditions for Determination of Antioxidant Activity by ABTS and DPPH Assays—A Practical Approach. Molecules.

[22] Platzer M., Kiese S., Herfellner T., Schweiggert-Weisz U., Miesbauer O., Eisner P. (2021). Common Trends and Differences in Antioxidant Activity Analysis of Phenolic Substances Using Single Electron Transfer Based Assays. Molecules.

[23] Slimestad R., Solheim H. (2002). Anthocyanins from Black Currants (*Ribes nigrum* L.). J. Agric. Food Chem..

[24] Paunović S.M., Mašković P., Nikolić M., Miletić R. (2017). Bioactive Compounds and Antimicrobial Activity of Black Currant (*Ribes nigrum* L.) Berries and Leaves Extract Obtained by Different Soil Management System. Sci. Hortic..

[25] Maatta K., Kamal-Eldin A., Törrönen R. (2001). Phenolic Compounds in Berries of Black, Red, Green, and White Currants (*Ribes* sp.). Antioxid. Redox Signal..

[26] Šimerdová B., Bobríková M., Lhotská I., Kaplan J., Křenová A., Šatínský D. (2021). Evaluation of Anthocyanin Profiles in Various Blackcurrant Cultivars over a Three-Year Period Using a Fast HPLC-DAD Method. Foods.

[27] Mattila P.H., Hellström J., Karhu S., Pihlava J.-M., Veteläinen M. (2016). High Variability in Flavonoid Contents and Composition between Different North-European Currant (*Ribes* spp.) Varieties. Food Chem..

[28] Kikas A., Rätsep R., Kaldmäe H., Aluvee A., Libek A.-V. (2020). Comparison of Polyphenols and Anthocyanin Content of Different Blackcurrant (*Ribes nigrum* L.) Cultivars at the Polli Horticultural Research Centre in Estonia. Agron. Res..

[29] Tian Y., Laaksonen O., Haikonen H., Vanag A., Ejaz H., Linderborg K., Karhu S., Yang B. (2019). Compositional Diversity among Blackcurrant (*Ribes nigrum*) Cultivars Originating from European Countries. J. Agric. Food Chem..

[30] Jaakola L. (2013). New Insights into the Regulation of Anthocyanin Biosynthesis in Fruits: Trends in Plant Science. Trends Plant Sci..

[31] Ma Y., Ma X., Gao X., Wu W., Zhou B. (2021). Light Induced Regulation Pathway of Anthocyanin Biosynthesis in Plants. Int. J. Mol. Sci..

[32] Parkar S.G., Redgate E.L., McGhie T.K., Hurst R.D. (2014). In Vitro Studies of Modulation of Pathogenic and Probiotic Bacterial Proliferation and Adhesion to Intestinal Cells by Blackcurrant Juices. J. Funct. Foods.

[33] Benvenuti S., Pellati F., Melegari M., Bertelli D. (2004). Polyphenols, Anthocyanins, Ascorbic Acid, and Radical Scavenging Activity of *Rubus*, *Ribes*, and *Aronia*. J. Food Sci..

[34] Rachtan-Janicka J., Ponder A., Hallmann E. (2021). The Effect of Organic and Conventional Cultivations on Antioxidants Content in Blackcurrant (*Ribes nigrum* L.) Species. Appl. Sci..

[35] Mikulic-Petkovsek M., Rescic J., Schmitzer V., Stampar F., Slatnar A., Koron D., Veberic R. (2015). Changes in Fruit Quality Parameters of Four *Ribes* Species during Ripening. Food Chem..

[36] Sarkar B., Kumar D., Sasmal D., Mukhopadhyay K. (2014). Antioxidant and DNA Damage Protective Properties of Anthocyanin-Rich Extracts from *Hibiscus* and *Ocimum*: A Comparative Study. Nat. Prod. Res..

[37] Nisca A., Ștefănescu R., Stegăruș D.I., Mare A.D., Farczadi L., Tanase C. (2021). Comparative Study Regarding the Chemical Composition and Biological Activity of Pine (*Pinus nigra* and *P. sylvestris*) Bark Extracts. Antioxidants.

[38] Lamuela-Raventós R.M. (2018). Folin–Ciocalteu Method for the Measurement of Total Phenolic Content and Antioxidant Capacity. Measurement of Antioxidant Activity & Capacity.

[39] Ainsworth E.A., Gillespie K.M. (2007). Estimation of Total Phenolic Content and Other Oxidation Substrates in Plant Tissues Using Folin–Ciocalteu Reagent. Nat. Protoc..

[40] Tanase C., Babotă M., Nișca A., Nicolescu A., Ștefănescu R., Mocan A., Farczadi L., Mare A.D., Ciurea C.N., Man A. (2023). Potential Use of *Quercus dalechampii* Ten. and *Q. frainetto* Ten. Barks Extracts as Antimicrobial, Enzyme Inhibitory, Antioxidant and Cytotoxic Agents. Pharmaceutics.

